# Epigenetics in pharmacogenes encoding metabolizing enzymes of second-generation antipsychotics used in schizophrenia and its clinical implications

**DOI:** 10.3389/fphar.2025.1611203

**Published:** 2025-09-04

**Authors:** Angel T. Alvarado, Amparo Iris Zavaleta, César Li-Amenero, María R. Bendezú, Jorge A. Garcia, Haydee Chávez, Juan J. Palomino-Jhong, Felipe Surco-Laos, Doris Laos-Anchante, Elizabeth J. Melgar-Merino, Mario Bolarte-Arteaga, Nesquen Tasayco-Yataco, Ricardo Pariona-Llanos

**Affiliations:** ^1^ Research Unit in Molecular Pharmacology and 4P Medicine, VRI, San Ignacio de Loyola University, Lima, Peru; ^2^ Molecular Biology Laboratory of the Faculty of Pharmacy and Biochemistry of the National University of San Marcos, Lima, Peru; ^3^ Victor Larco Herrera Hospital, Lima, Peru; ^4^ Faculty of Pharmacy and Biochemistry, San Luis Gonzaga National University, Ica, Peru; ^5^ Human Medicine, Continental University, Lima, Peru; ^6^ Human Medicine, Faculty of Health Sciences, Norbert Wiener University, Lima, Peru; ^7^ FIA, Peruvian University of Applied Sciences, Lima, Peru

**Keywords:** epigenetics, pharmacogenes, schizophrenia, antipsychotic drugs, clinical implications

## Abstract

Schizophrenia is a neuropsychiatric disorder caused by neurochemical alterations, non-genetic, genetic, epigenetic and environmental factors. Pharmacoepigenetics studies the relationship between epigenetic variability and response to drugs. The objective was to realize a descriptive review of the current state of knowledge on epigenetic molecular mechanisms in pharmacogenes encoding metabolizing enzymes of second-generation antipsychotics drugs used in schizophrenia and their clinical implications. A brief description of the pharmacogenes *CYP2D6, CYP1A2, CYP2C9, CYP2C19* and *CYP3A4*, enzymes and metabolism of second-generation antipsychotic drugs (SGAs) such as clozapine, olanzapine, risperidone, paliperidone and quetiapine was made. The central review was on the epigenetic molecular mechanisms of DNA methylation, histone methylation and acetylation of pharmacogenes, likewise, epigenetic changes due to enzyme-inducing drugs and SGAs, and their clinical implications, were described. Despite the limited scientific literature published on the epigenetics that regulate pharmacogenes, it has been shown that DNA methylation and histone trimethylation and acetylation are the main epigenetic mechanisms in pharmacogenes, alike, some enzyme-inducing drugs would promote epigenetic changes. This review has clinical implications for the medical-clinical care and treatment of schizophrenia.

## 1 Introduction

Schizophrenia is a neuropsychiatric disease that affects 1% of the general population and is one of the 10 leading causes of disability in developed countries. It has also been published that 5% of patients commit suicide, more than 30% of patients are potentially suicidal, and it is one of the main causes of suicide among young patients (Delphin et al., 2024). Hence, the importance of understanding how epigenetics, pharmacogenetics, and pharmacoepigenetics influence schizophrenia. Epigenetics is the science that studies the molecular mechanisms that modify DNA without altering the sequence of the genetic code, but contribute to hereditary phenotypic variations, and are essential for cells to remember past events, developmental alterations and external stimulation (Ocaña-Paredes et al., 2024; Smith et al., 2023; Fitz-James and Cavalli, 2022; Li, 2021; Srivastava et al., 2021; Zhang et al., 2020). Among the known epigenetic molecular mechanisms are DNA methylation of cytosine residues in CpG dinucleotides (cytosine-phosphate-guanine), histone modifications, processes mediated by non-coding RNAs (ncRNAs), such as small ncRNAs (sncRNAs) and long ncRNAs (lncRNAs) (Smigielski et al., 2020). While pharmacoepigenetics studies the relationship between epigenetic changes and response to drugs, helping to predict adverse drug reactions and their clinical efficacy (Smith et al., 2023; Lisoway et al., 2021).

Studying the pharmacogenetic and pharmacoepigenetic foundations of schizophrenia is crucial to understand how genetics, epigenetics, and environmental factors contribute to the evolution of the disease (Lisoway et al., 2021), also to understand how epigenetic molecular mechanisms influence the pharmacogenes *CYP2D6, CYP1A2, CYP2C9, CYP2C19* and *CYP3A4*, and how their modified allelic variants influence metabolic variability, plasma levels, and adverse drug reactions induced by antipsychotic drugs (Swathy and Banerjee, 2022; Cacabelos, 2020). In this context, pharmacogenetics and pharmacoepigenetics are the basis for the clinical science known as personalized or precision genomic medicine, which seeks to individualize the dose from the beginning of drug therapy, considering the genotype and phenotype profile according to ethnicity, mixed race, origin, sex and lifestyle, the purpose of which is to maintain plasma concentrations within the therapeutic range, minimizing adverse drug reactions, and optimizing the efficacy of the drug (Alvarado et al., 2022; Halbert, 2022; Imani et al., 2022; Hasanzad et al., 2021; Alvarado et al., 2019). To control the symptoms of schizophrenia, “typical” antipsychotic drugs or second-generation antipsychotic drugs (SGAs), also known as “atypical”, are used, which include clozapine, amisulpride, aripiprazole, asenapine, brexpiprazole, cariprazine, iloperidone, lurasidone, olanzapine, paliperidone, quetiapine, risperidone, sertindole, ziprasidone, and zotepine (Rognoni et al., 2021).

A search was conducted for published evidence in PubMed, MEDLINE, and Scopus databases. Optimizing the search with keywords that included “clozapine,” “olanzapine,” “risperidone,” “pharmacogenes,” and combinations with “epigenetic molecular mechanism,” “DNA methylation,” “histone methylation,” and “histone acetylation.” The search results were restricted to the last 25 years, so publications from 2000 to 10 April 2025, were retrieved. The descriptive review included articles with a focus on epigenetics in pharmacogenes that encode metabolizing enzymes of second-generation antipsychotics used in schizophrenia. All studies with a theme different from the inclusion criteria were excluded. For these considerations, the objective was to realize a descriptive review of the current state of knowledge on epigenetic molecular mechanisms in pharmacogenes encoding metabolizing enzymes of second-generation antipsychotics drugs used in schizophrenia and their clinical implications. We examined the pharmacogenetics of the *CYP2D6, CYP1A2, CYP2C9, CYP2C19*, and *CYP3A4* genes encoding their respective enzymes that metabolize SGAs such as clozapine, olanzapine, risperidone, paliperidone, and quetiapine. The epigenetic molecular mechanisms in pharmacogenes during the development of the individual, and by enzyme-inducing drugs, are described. In addition, this review analyzes the clinical implications of epigenetic change and its application in the clinical practice of schizophrenia.

## 2 Pharmacogenetics

Pharmacogenetics allows us to know the genotypes that predict metabolic phenotypes that influence plasma levels, therapeutic efficacy and adverse drug reactions (Kappel et al., 2024; Alvarado et al., 2022; Alvarado et al., 2021), hence the importance of analyzing the *CYP2D6, CYP1A2, CYP2C9, CYP2C19* and *CYP3A4* genes that code for their respective enzymes. Likewise, the metabolism of the main SGAs such as clozapine, olanzapine, risperidone, paliperidone and quetiapine are described (Ingelman-Sundberg and Sim, 2010; Ingelman-Sundberg et al., 2007).

### 2.1 Genes encoding SGA-metabolizing enzymes

The *CYP2D6* gene mapped to the long (q) arm of chromosome 22, region 13.2 (22q13.2) (Wielandt et al., 2022; Dorado et al., 2017; Zanger et al., 2004). The wild-type CYP2D6*1 allele generates the CYP2D6*1/*1 genotype that predicts normal metabolizers (NM). The main genotypes predicting intermediate metabolizers (IM) are CYP2D6*1/*19, CYP2D6*4/*10, CYP2D6*6/*10, CYP2D6*6/*17, CYP2D6*10/*10, CYP2D6*10/*19, CYP2D6*10/*41, CYP2D6*19/*41 and CYP2D6*49/*49; while CYP2D6*3/*3, CYP2D6*4/*4 and CYP2D6*5/*5 predict poor metabolizers (PM) (Rojas-Macetas et al., 2023; Alvarado et al., 2021). The gene encodes the protein cytochrome P450 2D6 (CYP2D6) that is expressed in the liver (it constitutes 2% of the total content of CYP 450), and in a lower percentage is found in the brain, where it could metabolize antipsychotics (Ur Rasheed et al., 2017; Peñas-Lledó and Llerena, 2014). This enzyme is responsible for metabolizing approximately 25% of the drugs used in clinical practice (Wielandt et al., 2022; Alvarado et al., 2021; Neyshaburinezhad et al., 2021).

The *CYP1A2* gene is located on the long (q) arm of chromosome 15, region 24.1 (15q24.1) (Zhou et al., 2009). The CYP1A2*1A/*1A genotype predicts NM and the CYP1A2*1C/*1C genotype predicts PM (Forster et al., 2021). This gene encodes the CYP1A2 protein which accounts for 8%–15% of the total CYP 450 content of hepatocytes (Al-Ahmad et al., 2017; Zhou et al., 2009). This enzyme participates in the hydroxylation or dealkylation of various drugs used clinically (Wielandt et al., 2022; Zhou et al., 2009).

The *CYP2C9* gene is mapped to the long (q) arm of chromosome 10, region 24 (10q24) and the wild-type allele CYP2C9*1 generates the CYP2C9*1/*1 genotype that predicts NM (Karnes et al., 2021; Céspedes-Garro et al., 2015). CYP2C9*1/*2, CYP2C9*2/*2 and CYP2C9*1/*3 genotypes predict IM (Alvarado et al., 2025; de Andrés et al., 2021); CYP2C9*2/*3, CYP2C9*3/*3, CYP2C9*5/*5 and CYP2C9*6/*6 predict PM (de Andrés et al., 2021; Karnes et al., 2021). This gene encodes the CYP2C9 enzyme, which is expressed in the liver and represents 18% of CYP 450 proteins and metabolizes approximately 15% of clinically used drugs (Wielandt et al., 2022; Johannessen Landmark et al., 2020; Alvarado et al., 2019).

The *CYP2C19* gene is located on the long arm (q) of chromosome 10, region 24.1 (10q24.1), and the wild allele CYP2C19*1 generates the CYP2C19*1/*1 genotype that predicts NM (Maruf et al., 2019; Yin and Miyata, 2011). CYP2C19*1/*2 predicts MI (de Andrés et al., 2021), CYP2C19*2/*2, CYP2C19*2/*3, CYP2C9*3/*3, CYP2C19*4/*4, CYP2C19*5/*5, CYP2C19*6/*6 and CYP2C19*7/*7 predict PM (Alvarado et al., 2023a; de Andrés et al., 2021). This gene encodes the CYP2C19 protein that metabolizes various drugs (Skadrić and Stojković, 2020; Bousman et al., 2019).

The *CYP3A4* gene is mapped to the long (q) arm of chromosome 7, region 22.1 (locus 7q22.1), and the wild-type allele CYP3A4*1.002 (previously known as CYP3A4*1A), generates the CYP3A4*1/*1 genotype that predicts normal metabolizers (NM) (Musyoka et al., 2024; Wielandt et al., 2022). CYP3A4*2/*2 is the genotype that predicts MI, while CYP3A4*3/*3, CYP3A4*20/*20 and CYP3A4*22/*22 genotypes predict poor metabolizers (Zhou et al., 2019; Apellániz-Ruiz et al., 2015). This gene encodes the CYP3A4 protein that is expressed in the liver (60% of total CYP450) and intestine (70% of total CYP450) (Alvarado et al., 2023b; Zhou et al., 2019). This enzyme is responsible for the oxidative metabolism of 30% of drugs in clinical use, therefore, the allelic variants that encode it can modify the pharmacokinetic parameters, be involved in the toxicity and clinical outcome of pharmacological treatment (Collins and Wang, 2022; Wielandt et al., 2022).

### 2.2 Metabolism of the principal SGAs

Regarding the metabolism of the six SGAs, it has been described that aripiprazole is metabolized by phase I of hydroxylation and dehydrogenation. Aripiprazole is converted to 4-hydroxy-aripiprazole by CYP3A4/CYP2D6 and subsequently metabolized by glucuronidation; dehydrogenation produces the active metabolite dehydroaripiprazole (Caccia, 2007). The metabolite dehydroaripiprazole undergoes two reactions, the first reaction is hydroxylation to generate 4-hydroxy-dehydroaripiprazole, this process occurs through the action of CYP3A4/CYP2D6, subsequently 4-hydroxy-dehydroaripiprazole is metabolized by glucuronidation; The second reaction is by N-dealkylation at the piperazinyl nitrogen, originating dehydroaripiprazole acid and 1-(2,3-dichlorophenyl)-piperazine, this process is generated with the participation of the CYP3A4 isoenzyme (Caccia, 2007). The CYP3A4 isoenzyme participates in a third metabolic pathway of N-dealkylation of aripiprazole at the piperazinyl nitrogen level, producing butoxy-3,4-dihydro-1H-quinolin-2-one acid and 1-(2,3-dichlorophenyl)-piperazine. The metabolite 1-(2,3-dichlorophenyl)-piperazine is converted into p-hydroxy-(2,3-dichlorophenyl)-piperazine with the participation of CYP2D6, and subsequently this last metabolite undergoes phase II of glucuronidation, for this, UDP-glucuronosyl transferase (UGT) transfers the glucuronic group from UDP-α-D-glucuronic acid (UDPGA) to generate the metabolite O-β-glucuronide of (2,3-dichlorophenyl)-piperazine (Caccia, 2007).

Clozapine is a tricyclic dibenzodiazepine derivative that is metabolized by phase I N-demethylation by CYP1A2 and CYP3A4 to generate the active metabolite norclozapine or N-desmethylclozapine (Kappel et al., 2024; Albitar et al., 2020), by hydroxylation with the participation of CYP2D6, CYP2C19 and CYP3A4, hydroxy-clozapine is formed. Clozapine N-oxidation generates the clozapine N-oxide metabolite, and the isoenzymes CYP3A4, CYP3A5, CYP1A2, CYP2C8 and CYP2C19 participate in this metabolic reaction. In phase II of N-glucuronidation, UDP-glucuronosyl transferase 1A3 (UGT1A3) transfers the glucuronic acid group from UDP-α-D-glucuronic acid (UDPGA) to generate the clozapine N-glucuronide metabolite (Hiemke et al., 2018; Mauri et al., 2018).

Olanzapine is a thienobenzodiazepine derivative that is metabolized by phase I into 4′-N-desmethylolanzapine and 7-hydroxyolanzapine by CYP1A2. CYP2D6, CYP3A4, and CYP2C9 convert olanzapine to 2-hydroxymethylolanzapine; CYP2D6 and flavin-containing monooxygenase 3 (FMO3) produce olanzapine N-oxide, all of which have low pharmacological activity (Kolli et al., 2023; Soria-Chacartegui et al., 2021). By the phase II reaction, olanzapine is directly conjugated (N-glucuronidation) with the participation of UDP-glucuronosyl transferase 1A4 (UGT1A4) and UGT2B10, which catalyzes the transfer of the glucuronic acid group from UDP-α-D-glucuronic acid (UDPGA), forming the metabolite 10-N-glucuronide of olanzapine (Kolli et al., 2023; Rojas-Macetas et al., 2023; Soria-Chacartegui et al., 2021).

Risperidone is a benzisoxazole derivative metabolized by phase I into (+)-9-hydroxyrisperidone (paliperidone) catalyzed by CYP2D6, and to a lesser extent it is metabolized by CYP3A4 forming (−)-9-hydroxyrisperidone (Soria-Chacartegui et al., 2021; Spina et al., 2020). A fraction of the drug is metabolized by N-dealkylation with the participation of CYP3A4 and CYP3A5, originating inactive metabolites (Soria-Chacartegui et al., 2021), the hydroxylated metabolites are then metabolized by phase II conjugation with UDP-glucuronosyl transferase (UGT) which transfers the glucuronic acid group from UDP-α-D-glucuronic acid (UDPGA) to the drug to form the O-β-glucuronide metabolite of risperidone which is eliminated in the urine (Rojas-Macetas et al., 2023). The half-life (t_1/2_) of risperidone is 3 h, and the active metabolite is between 20 and 24 h (Soria-Chacartegui et al., 2021). Conjugated and inactive metabolites are eliminated 70% in the urine and 14% in the bile; in cases of liver and kidney dysfunction, dose adjustment is required (Schoretsanitis et al., 2017).

Paliperidone is a metabolite of risperidone and chemically is a benzoisoxazole derivative (Vermeir et al., 2008) which is metabolized by phase I oxidation by CYP3A4 and minimally by CYP2D6 (Kanu et al., 2024). In normal metabolizers (NM), inducers (carbamazepine and others) of CYP3A4 and ABCB1 increase metabolism and decrease the plasma level of the drug (de Leon, 2020). The elimination half-life (t_1/2_) is 23–30 h, allowing a daily dose to be administered to maintain therapeutic plasma levels (Mauri et al., 2018; Vermeir et al., 2008). 60% is excreted unchanged in the urine and 10% in the bile (Mauri et al., 2018).

Quetiapine is a dibenzothiazepine that undergoes three phase I metabolic reactions: CYP3A4 metabolizes 89% of the quetiapine dose forming norquetiapine or N-desalkyl quetiapine, which is further metabolized by CYP2D6 to form 7-hydroxy N-alkyl quetiapine, this metabolite is converted into an inactive molecule by the action of the enzyme catechol O-methyltransferase (COMT). The second reaction is generated by the action of CYP2D6, converting quetiapine into 7-hydroxyquetiapine, which is biotransformed by COMT into an inactive metabolite. A third metabolic pathway activated by CYP2C9, CYP2C19, CYP1A2 and CYP3A5 biotransforms quetiapine into an inactive metabolite (Zubiaur et al., 2021; Kim et al., 2016).

## 3 General epigenetic molecular mechanisms

### 3.1 DNA methylation

**FIGURE 1:**
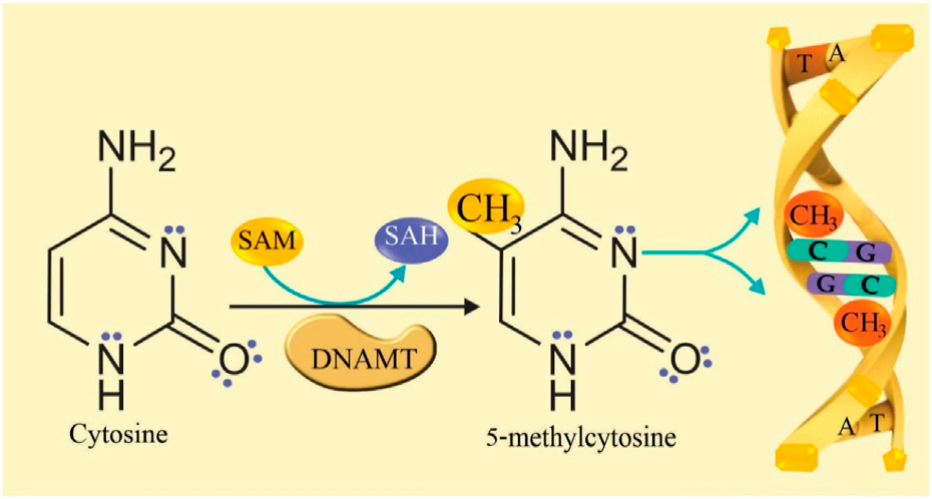
DNA methylation reaction mechanism. The enzyme DNA methyltransferase (DNMT) transfers a methyl group from S-adenosylmethionine (SAM) to C-5 of cytosine, forming 5-methylcytosine (5 mC). SAM is converted to S-adenosylhomocysteine (SAH).

DNA methylation is a normal mechanism that is generated in cytosine and in specific loci, and regulates gene transcription by direct or indirect process, directly prevents the binding of transcription factors in short DNA sequences in gene promoters, and indirectly interferes with the recruitment of corepressors by the action of methyl-CpG binding proteins (MBP) (Fitz-James and Cavalli, 2022; Li and Tollefsbol, 2021). DNA methylation occurs when DNA methyltransferase (DNMT) transfers a methyl group from S-adenosylmethionine (SAM) to C-5 of the cytosine ring that is linked to guanine (CpG) forming 5-methylcytosine (5 mC), and SAM is converted to S-adenosylhomocysteine (SAH), which loses adenosine to form homocysteine. Ten-eleven translocation (TET) enzymes subsequently convert 5mC to 5-hydroxymethylcytosine (5hmC), this occurs in the sequence of the dinucleotide CpG (Cytosine-Guanine) or CpHpG (where H can be A, T, C). The conversion occurs in the 5′to 3′direction of DNA during replication where a cytosine and a guanine are adjacent (Li and Tollefsbol, 2021; Richa and Sinha, 2014). The reaction mechanism of DNA methylation is proposed in Figure 1.

### 3.2 Histone methylation

**FIGURE 2:**
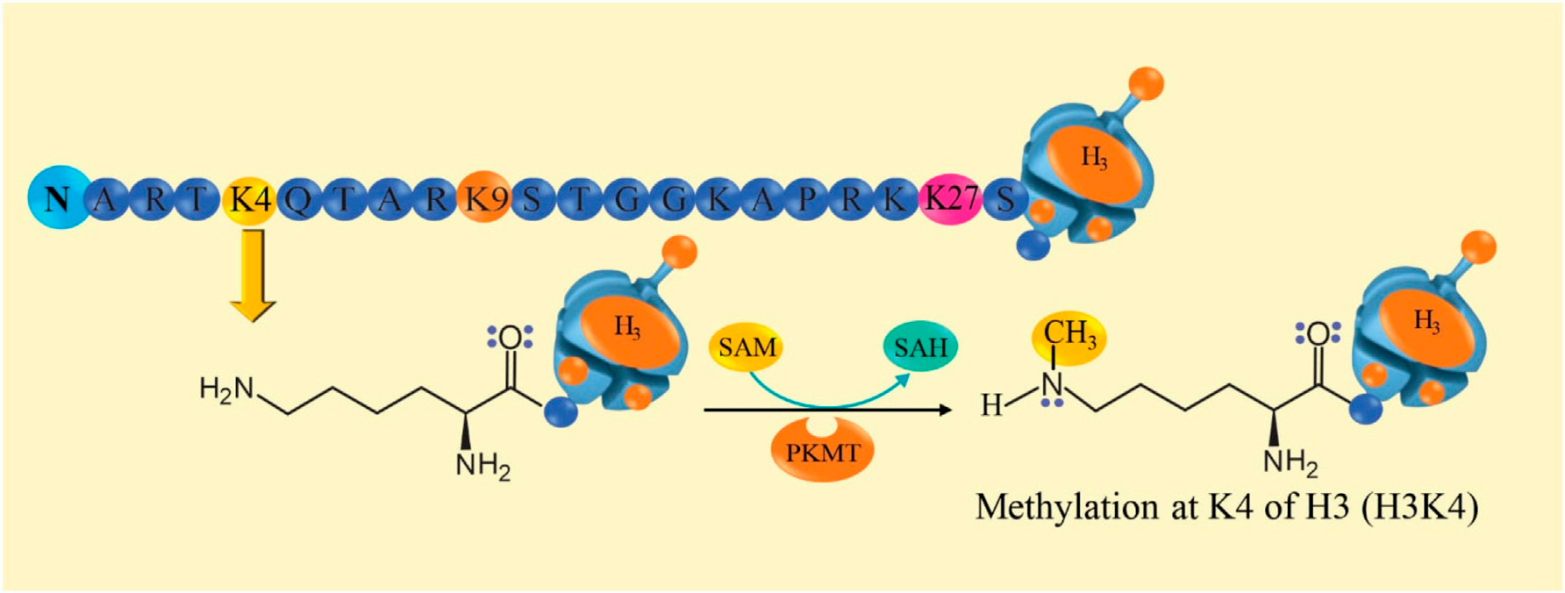
Histone methylation reaction mechanism. At the top, an amino acid sequence of the long tail of histone 3 (H3) is observed, with lysine 4 (K4) being susceptible to methylation. The mechanism of methylation is proposed: the histone lysine methyltransferase (PKMT) transfers a methyl group from S-adenosylmethionine (SAM) to the amino terminal group of histones 3 K4 (H3K4), this alters the binding of the histone to DNA. SAM is converted to S–adenosylhomocysteine (SAH).

Histones (H) are proteins that package DNA into nucleosomes, are found in chromosomes (Strahl and Allis, 2000; Liu et al., 2023) and regulate gene expression (Strahl and Allis, 2000). This molecule experiment methylation and acetylation reactions at specific amino acid residues, generating suppression or activation of transcription (Strahl and Allis, 2000). Methylation, dimethylation or trimethylation of lysine 9 (K9) of histone 3 (H3K9, H3K9me2 or H3K9me3), di- or tri-methylation of K27 of H3 (H3K27me2 or H3K27me3) in the promoter regions of genes and transcription start sites, they are epigenetic marks that produce suppression of gene transcription and, therefore, the genes that code for their respective proteins or enzymes are not expressed (Wang et al., 2008; Strahl and Allis, 2000; Abu-Hanna et al., 2022; Duan et al., 2022). Dimethylation of K4 (H3K4me2) (Wang and Helin, 2025), tri-methylation of K4 on H3 (H3K4me3) (Park et al., 2015), di- or tri-methylation at K36 of H3 (H3K36me2 or H3K36me3) (Wang et al., 2008; Duan et al., 2022) activate gene transcription and, therefore, the gene is more likely to be expressed and encode enzymes or proteins with greater activity, but it is not a unique and absolute signal of activation since there are other activating factors (Strahl and Allis, 2000; Park et al., 2015; Zhang et al., 2019; Liu et al., 2023; Wang and Helin, 2025). Histone methylation is a molecular mechanism that can alter the structure of chromosomes. This mechanism is catalyzed mainly by histone methyltransferase (HMT). These are two enzymes, the first is histone lysine methyltransferase (KMT) and depending on the sequence of the catalytic domain they can be SET and non-SET type, the SET domain includes SUV39H1/2, G9a, GLP, SMYD, SETDB1, EZH2 and others. The second HMT is the protein arginine methyltransferase (PRMT) which are of two classes, the first class consists of PRMT1, PRMT3, PRMT4, PRMT6 and PRMT8, responsible for the monomethyl arginine reaction (Guccione et al., 2021; Saha and Muntean, 2021; Torcal Garcia and Graf, 2021; Barghout et al., 2022). The amino acid sequence of the N-terminus of histone 3 is schematically represented in Figure 2 for illustrative purposes (not an accurate representation of the sequence): alanine (A), arginine (R), threonine (T), lysine (K4), glutamine (Q), threonine (T), alanine (A), arginine (R), lysine (K9), serine (S), threonine (T), glycine (G), glycine (G), lysine (K), alanine (A), proline (P), arginine (R), lysine (K), glutamine (Q), leucine (L), alanine (A), threonine (T), lysine (K), alanine (A), alanine (A), arginine (R), lysine (K27), serine (S), alanine (A), and proline (P). K4 and K27 are susceptible to methylation and K9 is susceptible to acetylation (Strahl and Allis, 2000). The reaction mechanism of histone methylation is proposed in Figure 2.

### 3.3 Histone acetylation

**FIGURE 3:**
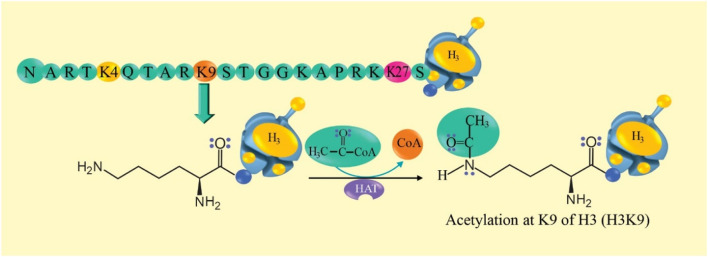
Histone acetylation reaction mechanism. At the top, an amino acid sequence of the long tail of histone 3 (H3) is observed. Lysine 9 (K9) of H3 is susceptible to the acetylation reaction. In the reaction, it is observed that histone acetyl transferase (HAT) transfers an acetyl group from “acetyl-CoA” to K9 of H3 (H3K9ac) and this could activate gene transcription.

Acetylation of the lysine residue of histones is the main mechanism (Li and Tollefsbol, 2021; Duan et al., 2022). Histone acetyl transferase (HAT) transfers an acetyl group from “acetyl-CoA” to histone lysine, which causes structural changes in the nucleosome and activation of transcription, while histone lysine deacetylase inhibitors deacetylate histones (HDAC) and repress gene transcription, this establishes a balance (Sun et al., 2015; Narita et al., 2019; Liu et al., 2023). Histones H3 and H4 are characterized by a long tail that protrudes from a specific position in the nucleosome core (Liu et al., 2023). Lysine (K9) acetylation of histone 3 (H3K9ac) has an activating effect (Strahl and Allis, 2000). The reaction mechanism of histone acetylation is proposed in Figure 3.

## 4 Pharmacoepigenetics

Pharmacoepigenetics studies the relationship between epigenetic variability and drug response, which could help with personalized drug treatment (Smith et al., 2023).

### 4.1 Epigenetic molecular mechanisms in pharmacogenes

Pharmacoepigenetic mechanisms such as DNA methylation, post-translational modifications of histones and regulation of microRNAs do not alter the nuclear DNA sequence but affect gene transcription that is expressed in hereditary phenotypic changes that alter the pharmacological response (Li, 2021; Recillas-Targa, 2022). Epigenetic changes in CYP genes are generated during the human growth process (from neonate to adulthood) (Thakur et al., 2021). In each human being and individually, epigenetic changes are generated by two factors, the first by liver dysfunction, which leads to a decrease in the metabolic activity of enzymes (Diep et al., 2017; Bao et al., 2020; Thakur et al., 2021), and the second individual factor is due to pharmacological treatment with enzyme-inducing drugs, which increase the expression of the enzymes and decrease the plasma levels of the drugs, resulting in therapeutic failure (Hakkola et al., 2020; Jin and Zhong, 2023).

#### 4.1.1 DNA methylation in pharmacogenes

Helsby and Burns (2012) observed that the 3′-untranslated region (3′UTR) of the *CYP2C19* gene contains two recognition sites (MRE) for the non-coding RNA microRNA and is susceptible to methylation generating suppression of gene translation. The tissue used was Human Hepatoma HepG2 Cells. In another study, DNA methylation of the *CYP2C19* gene was confirmed after using 5-aza-2′-deoxycytidine (5azaDC) as a methyltransferase inhibitor, but the existence of other, yet unidentified, epigenetic regulators of *CYP2C19* transcription was also suggested (Burns et al., 2018). Habano et al. (2015) described epigenetic methylation changes in DNA of *CYP2D6, CYP1A2* and *CYP2C19* genes, additionally methylation was evidenced in CpG sites in *CYP2C19* genes. These epigenetic changes were observed in fetal liver tissue (NLF), adult small intestine (NSI) tissue, and three hepatoma cell lines (HepG2, HuH7, and JHH1).

In *CYP3A4* genes, methylation occurs in the DNA promoter region, affecting the binding of RNA polymerase and transcription factors that initiate gene transcription. This study was performed in pediatric and prenatal human liver tissue (Vyhlidal et al., 2016). In another study, methylation was observed in CpG dinucleotides in the promoter region of the *CYP3A4* gene and in the body of the *CYP2D6* gene (Shi et al., 2017). By induced epimutation on the *CYP2D6* gene, cytosine (C) is lost from position 188 and is replaced by thymine (T) in exon 1 (C>T), giving rise to the CYP2D6*10 allele that expresses low-activity enzymes, which could explain the individual differences in pharmacokinetic parameters and provide new knowledge for the personalized treatment (Yingyuan et al., 2023). Table 1 describes the epigenetic molecular mechanism in pharmacogenes DNA and its clinical implications.

**TABLE 1:** Epigenetic molecular mechanism in pharmacogenes DNA.

Author	Pharmacogenes	Epigenetic molecular mechanism	Conclusions and their importance
Helsby and Burns. (2012)	*CYP2C19*	Methylation in the promoter region of DNA.	It causes silencing in one of the *CYP2C19* alleles, affecting the hepatic expression of the CYP2C19 enzyme
Habano et al. (2015)	*CYP2D6* *CYP1A2* *CYP2C19*	Methylation of the exons and the 5′regulatory region (5′UTR) of the *CYP2D6, CYP1A2* and *CYP2C19* genes	The DNA methylation mechanism silences one of the alleles of the genes studied, decreasing the expression of the enzymes, and this contributes to inducing adverse drug reactions
Vyhlidal et al. (2016)	*CYP3A4*	Methylation occurs in the promoter region of DNA.	Changes occur in the expression of mRNA for its enzyme
Shi et al. (2017)	*CYP2D6* *CYP3A4*	DNA methylation in the body of the *CYP2D6* gene CpG dinucleotide methylation in the promoter region of the *CYP3A4* gene	The CYP2D6 enzyme could decrease its functional activity and induce risperidone-induced adverse reactions

#### 4.1.2 Histone methylation of pharmacogenes

In experimental studies carried out with tissues, methylation has been observed in pharmacogene histones. The study reported by He et al. (2016) has described methylation in the long tail of histone H3 of the *CYP3A4* gene. In another investigation carried out by Park et al. (2015) it has been described that the promoter region of the *CYP2D6* gene contains 12 CpG dinucleotides (cytosine-phosphate-guanine) that are methylated, this has been observed in hepatocytes derived from human embryonic stem cells (hESC-Hep) with a frequency of 96.3%, the body region contains 32 CpG dinucleotides and the methylation frequency was 45.5% in human primary hepatocytes (hPH) and 90.3% in hESC-Hep. The *CYP1A2* gene contains 10 CpG dinucleotides in the promoter region and is fully methylated in hPH and hESC-Hep; the body region of the gene contains 20 CpG dinucleotides, and they are methylated 40.0% and 76.7% in hPH and hESC-Hep, respectively. Table 2 summarizes the epigenetic molecular mechanism in pharmacogenomic histones, the conclusions and their importance.

**TABLE 2:** Epigenetic molecular mechanism in pharmacogene histones and its clinical importance.

Author	Pharmacogenes	Epigenetic molecular mechanism	Conclusions and their importance
Kacevska et al. (2012)	*CYP3A4*	CpG hypermethylation of the *CYP3A4* regulatory region	Hypermethylation of the *CYP3A4* gene results in repression of gene transcription and therefore the CYP3A4 enzyme is not expressed. This allows us to understand interindividual differences in drug response
He et al. (2016)	*CYP3A4*	Di-methylation at K4 of H3 (H3K4me2) activates gene transcription Tri-methylation at K27 of H3 (H3K27me3) suppresses gene transcription	Methylation may affect *CYP3A4* mRNA expression in adult liver Epigenetic changes in H3K27me3 and H3K4me2 are associated with development from conception to adulthood
Park et al. (2015)	*CYP2D6* *CYP1A2*	Tri-methylation at K4 of H3 (H3K4me3) of the *CYP2D6* promoter region in hPH. Tri-methylation at K27 of histone-3 (H3K27me3) in hESC-Hep suppresses *CYP1A2* transcription Tri-methylation of K4 on H3 (H3K4me3) in the *CYP1A2* body region activates transcription in hPH.	The limited expression of *CYP2D6* and *CYP1A2* genes in hESC-Hep is modulated by epigenetic regulatory factors such as DNA methyltransferases (DNMTs)

H3K27me3: tri-methylation at lysine 27 (K27) of histone-3 (H3); H3K4me2: di-methylation at lysine 4 (K4) of histone-3 (H3); hESC-Hep: human embryonic stem cells; hPH: human primary hepatocytes; CpG: dinucleotides.

**FIGURE 4:**
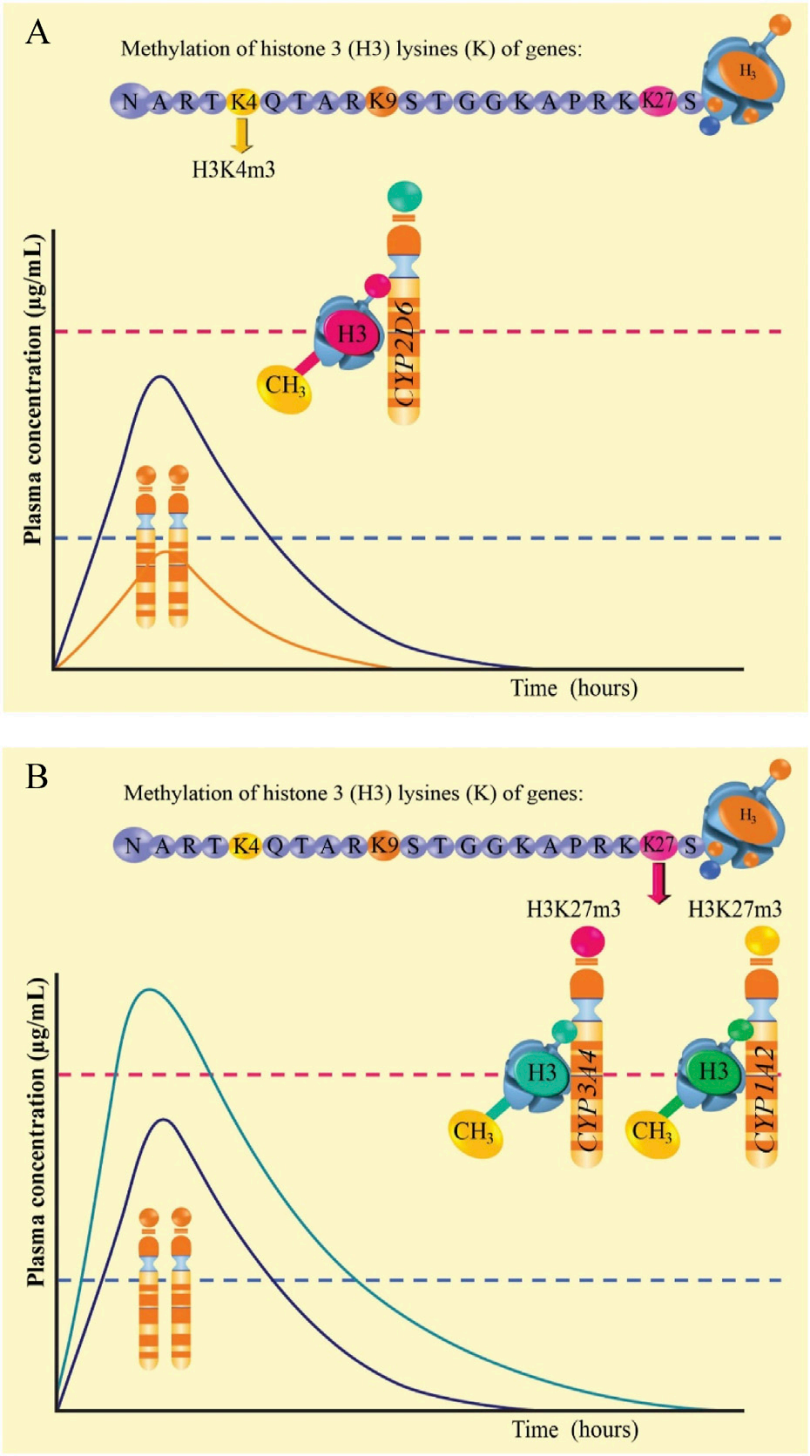
Graphic representation of methylation of histone 3 lysine 4 of the *CYP2D6* gene **(A)** and methylation of histone 3 lysine 27 of the *CYP3A4* and *CYP1A2* genes** (B).**

In Figures 4A,B it is observed the amino acid sequence of the long tail of histone 3 of the *CYP2D6*, *CYP3A4,* and *CYP1A2* genes. In 4A, trimethylation is proposed in lysine 4 (K4) of histone 3 of the *CYP2D6* gene (H3K4m3), generating major genetic transcription and, therefore, active CYP2D6 enzymes would be expressed. This would generate major metabolism and decrease in the plasma level of the drug, even below the minimum effective concentration (below the dashed blue line and orange curve), at the same time, a curve (purple) is indicated within the therapeutic range in patients with normal genotype and phenotype. Figure 4B shows trimethylation at K27 of histone 3 of the *CYP3A4* (H3K27m3) and *CYP1A2* (H3K27m3) genes. This would generate scares or null metabolism, increasing the plasma level of the drug beyond the minimum toxic concentration (above the dashed red line and green curve green), compared to the curve (purple) which is within the therapeutic range.

### 4.2 Epigenetic molecular mechanisms by enzyme-inducing drugs

Carbamazepine, phenytoin, rifampicin, and others induce *CYP3A4* transcription resulting in increased enzymes (Hakkola et al., 2020), which leads to increased metabolism and decreased plasma levels of aripiprazole, so it may be necessary to double the dose of aripiprazole (DeLeon et al., 2004).

Rifampicin has been reported to activate histone H3 acetylation, activate histone 3-lysine-4 tri-methylation (H3K4me3), and decrease histone-3-lysine-27 tri-methylation (H3K27me3) at promoter regions, which contributes to the induction of *CYP3A4* mRNA expression (Yan et al., 2017). Long non-coding RNAs (lncRNAs) are involved in the induced expression of CYP450 enzymes during treatment with inducing drugs such as rifampicin and phenobarbital (Chen et al., 2018), in another study, hepatocyte nuclear factor 4 alpha antisense RNA 1 (HNF4A-AS1) was reported to induce histone 3 tri-methylation generating H3K4me3 and H3K27me3 (Wang et al., 2021).

### 4.3 Epigenetic molecular mechanisms induced by drugs used in schizophrenia

It has been studied that some second-generation antipsychotic drugs such as clozapine, risperidone, olanzapine and quetiapine can induce epigenetic demethylation changes (Swathy and Banerjee, 2017). Table 3 summarizes the various studies that describe drug-induced epigenetic molecular mechanisms in schizophrenia.

**TABLE 3:** Epigenetic molecular mechanism induced by second-generation antipsychotic drugs and its importance.

Author	Study type	Drug	Epigenetic molecular mechanism	Conclusions and their importance
Dong et al. (2008)	Experimental study in mice	Clozapine	Clozapine increases promoter-associated H3K9 and H3K14 acetylation and causes demethylation of RELN and GAD67 gene promoters (hypermethylated) in striatal GABAergic neurons	DNA demethylation could mediate hypomethylation of the promoter of GABAergic genes (inhibited in psychosis), increasing their expression, alleviating GABA deficiency pathology in schizophrenia and bipolar disorder
Guidotti et al. (2011)	Experimental study in mice	Clozapine and olanzapine	Activates demethylation of the GAD67 promoter in GABAergic neurons	Activating DNA demethylation with clozapine and olanzapine with VPA (histone deacetylase inhibitor, HDAC) may be considered a promising therapeutic strategy to normalize GABAergic promoter hypermethylation and decreased GABAergic gene expression detected in postmortem brains of patients with schizophrenia and bipolar disorder
Melka et al. (2014)	Experimental study in rats	Olanzapine	Olanzapine induces DNA methylation of genes in the dopaminergic pathway that encodes transporter proteins, receptors, nervous system development, and hippocampal functions	Epigenetic changes could be the cause of the improvement in symptoms and could also explain certain adverse effects
Li et al. (2004)	Experimental study in mice	Risperidone	Risperidone blocks DR2 to induce histone 3 phosphoacetylation (H3pS10-acK14) through cAMP-dependent PKA and postsynaptic NMDA receptor signaling H3pS10-acK14 is overexpressed in the striatum	Histone modifications and chromatin structure in striatal neurons are dynamically regulated by dopaminergic and glutamatergic stimuli
Rami et al. (2022)	Experimental study in mice	Risperidone	Methylation at the cytosine-phosphate-guanine 2 (CpG2) site of DRD2 in risperidone-treated mice	Risperidone generates low methylation in DRD2 of PFC and AMY, which improves their expression levels

AMY, amygdala; PFC, prefrontal cortex; DRD2, dopaminergic receptor type 2; H3K9, histone 3 lysine 9; H3K14, histone 3 lysine 14; RELN, RELN, gene encoding the reelin protein; GAD67, glutamate decarboxylase 67; PKA, protein kinase A; cAMP, adenosine 3′,5′-cyclic monophosphate; NMDA, N-methyl-D-aspartate.

## 5 Clinical implications of epigenetic mechanisms

The epigenetic mechanisms of histone trimethylation and DNA methylation in *CYP3A4, CYP2D6, CYP1A2* and *CYP2C9* genes cause silencing in one of the alleles, which would explain the presence of genotypes that predict metabolic phenotypes associated with a higher risk of adverse drug reactions and therapeutic failure in patients receiving second-generation antipsychotic drugs. Therefore, a strategy to demethylate the DNA of GABAergic promoters and dopaminergic receptor type 2 (DR2) would be to use clozapine, olanzapine and risperidone associated with valproic acid (histone deacetylase inhibitor) to ensure the efficacy of the drug in patients with schizophrenia and bipolar disorder. These findings have clinical implications for schizophrenia, given that specialist physicians have scientific evidence for medical care, their daily clinical practice, and consider it before initiating pharmacological treatment.

The results of this descriptive review have limitations, which may lead to bias. The main one is the limited or scarce scientific literature published on pharmacoepigenetics in schizophrenia, the limited specific literature on epigenetic changes in genes encoding second-generation antipsychotic drug-metabolizing enzymes, and articles with limited methodology (small sample size, *in vitro* study of different tissue types, and experimental animal models). However, this research contributes to updating and synthesizing the pharmacoepigenetic knowledge published to date, while also generating scientific evidence for conducting prospective longitudinal cohort studies, observational studies in humans, standardized epigenetic clinical trials, and integrated pharmacoepigenetic-pharmacokinetic study.

## 6 Conclusion and future perspectives

Based on the review of published scientific evidence, it is concluded that DNA methylation, histone methylation and acetylation are the most frequent epigenetic mechanisms in the *CYP3A4, CYP2D6*, *CYP1A2* and *CYP2C19* pharmacogenes. At the same time, the use of enzyme-inducing drugs can also generate epigenetic changes.

These epigenetic changes could explain the expression of intra- and interindividual allelic variants and, therefore, influence the efficacy and safety of second-generation antipsychotic drugs used in schizophrenia. On the other hand, further epigenetic studies are required on pharmacogenes and on a greater number of biological samples from patients with schizophrenia, to corroborate the published research that has been summarized in this descriptive review.

These studies are also relevant for medical specialists, who will have an additional tool to consider epigenetic mechanisms as a possible factor influencing drug treatment. At the same time, this study will contribute to the scientific evidence base for promoting the inclusion of epigenetic data in a Pharmacogenomic Guide and implementing personalized or precision genomic medicine in healthcare systems, with the goal of improving the quality of life of patients with schizophrenia.

## References

[B1] Abu-Hanna J. Patel J. A. Anastasakis E. Cohen R. Clapp L. H. Loizidou M. (2022). Therapeutic potential of inhibiting histone 3 lysine 27 demethylases: a review of the literature. Clin. Epigenet 14, 98. 10.1186/s13148-022-01305-8 35915507 PMC9344682

[B2] Al-Ahmad M. M. Amir N. Dhanasekaran S. John A. Abdulrazzaq Y. M. Ali B. R. (2017). Genetic polymorphisms of cytochrome P450-1A2 (CYP1A2) among emiratis. PloS one 12 (9), e0183424. 10.1371/journal.pone.0183424 28934216 PMC5608188

[B3] Albitar O. Harun S. N. Zainal H. Ibrahim B. Sheikh Ghadzi S. M. (2020). Population pharmacokinetics of clozapine: a systematic review. Biomed. Res. Int. 2020, 9872936. 10.1155/2020/9872936 31998804 PMC6970501

[B4] Alvarado Á. T. Muñoz A. M. Loja B. Miyasato J. M. García J. A. Cerro R. A. (2019). Study of the allelic variants CYP2C9*2 and CYP2C9*3 in samples of the Peruvian mestizo population. Biomedica 39 (3), 601–610. 10.7705/biomedica.4636 31584773 PMC7357368

[B5] Alvarado A. T. Ybañez-Julca R. Muñoz A. M. Tejada-Bechi C. Cerro R. Quiñones L. A. (2021). Frequency of CYP2D6*3 and *4 and metabolizer phenotypes in three mestizo Peruvian populations. Pharmacia 68 (4), 891–898. 10.3897/pharmacia.68.e75165

[B6] Alvarado A. T. Paredes G. García G. Morales A. Muñoz A. M. Saravia M. (2022). Serum monitoring of carbamazepine in patients with epilepsy and clinical implications. Pharmacia 69 (2), 401–406. 10.3897/pharmacia.69.e82425

[B7] Alvarado A. T. Muñoz A. M. Varela N. Sullón-Dextre L. Pineda M. Bolarte-Arteaga M. (2023a). Pharmacogenetic variants of CYP2C9 and CYP2C19 associated with adverse reactions induced by antiepileptic drugs used in Peru. Pharmacia 70 (3), 603–618. 10.3897/pharmacia.70.e109011

[B8] Alvarado A. T. Muñoz A. M. Ybañez R. O. Pineda M. Tasayco N. Bendezú G. (2023b). SLCO1B1 and CYP3A4 allelic variants associated with pharmacokinetic interactions and adverse reactions induced by simvastatin and atorvastatin used in Peru: clinical implications. J. Pharm. Pharmacogn. Res. 11 (6), 934–952. 10.56499/jppres23.1686_11.6.934

[B9] Alvarado A. T. Zavaleta A. I. Li-Amenero C. Bendezú M. R. Garcia J. A. Chávez H. (2025). Role of pharmacogenomics for prevention of hypersensitivity reactions induced by aromatic antiseizure medications. Front. Pharmacol. 16, 1640401. 10.3389/fphar.2025.1640401 40873549 PMC12378293

[B10] Apellániz-Ruiz M. Inglada-Pérez L. Naranjo M. E. Sánchez L. Mancikova V. Currás-Freixes M. (2015). High frequency and founder effect of the CYP3A4*20 loss-of-function allele in the Spanish population classifies CYP3A4 as a polymorphic enzyme. Pharmacogenomics J. 15 (3), 288–292. 10.1038/tpj.2014.67 25348618

[B11] Bao Y. Wang P. Shao X. Zhu J. Xiao J. Shi J. (2020). Acetaminophen-induced liver injury alters expression and activities of cytochrome P450 enzymes in an age-dependent manner in mouse liver. Drug Metab. Dispos. 48 (5), 326–336. 10.1124/dmd.119.089557 32094214 PMC7153563

[B12] Barghout S. H. Machado R. A. C. Barsyte-Lovejoy D. (2022). Chemical biology and pharmacology of histone lysine methylation inhibitors. Biochim. Biophys. Acta Gene Regul. Mech. 1865 (6), 194840. 10.1016/j.bbagrm.2022.194840 35753676

[B13] Bousman C. A. Menke A. Müller D. J. (2019). Towards pharmacogenetic-based treatment in psychiatry. *J. Neural Transm*. (Vienna) 126 (1), 1–3. 10.1007/s00702-018-01968-9 30673860

[B14] Burns K. E. Shepherd P. Finlay G. Tingle M. D. Helsby N. A. (2018). Indirect regulation of CYP2C19 gene expression via DNA methylation. Xenobiotica 48 (8), 781–792. 10.1080/00498254.2017.1372648 28840784

[B15] Cacabelos R. (2020). Pharmacogenomics of cognitive dysfunction and neuropsychiatric disorders in dementia. Int. J. Mol. Sci. 21 (9), 3059. 10.3390/ijms21093059 32357528 PMC7246738

[B16] Caccia S. (2007). N-dealkylation of arylpiperazine derivatives: disposition and metabolism of the 1-aryl-piperazines formed. Curr. Drug Metab. 8 (6), 612–622. 10.2174/138920007781368908 17691920

[B17] Céspedes-Garro C. Fricke-Galindo I. Naranjo M. E. Rodrigues-Soares F. Fariñas H. de Andrés F. (2015). Worldwide interethnic variability and geographical distribution of CYP2C9 genotypes and phenotypes. Expert Opin. Drug Metab. Toxicol. 11 (12), 1893–1905. 10.1517/17425255.2015.1111871 26595139

[B18] Chen L. Bao Y. Piekos S. C. Zhu K. Zhang L. Zhong X. B. (2018). A transcriptional regulatory network containing nuclear receptors and long noncoding RNAs controls basal and drug-induced expression of cytochrome P450s in HepaRG cells. Mol. Pharmacol. 94 (1), 749–759. 10.1124/mol.118.112235 29691280 PMC5988030

[B19] Collins J. M. Wang D. (2022). Regulation of CYP3A4 and CYP3A5 by a lncRNA: a potential underlying mechanism explaining the association between CYP3A4*1G and CYP3A metabolism. Pharmacogenet Genomics 32 (1), 16–23. 10.1097/FPC.0000000000000447 34320606 PMC8578198

[B20] de Andrés F. Altamirano-Tinoco C. Ramírez-Roa R. Montes-Mondragón C. F. Dorado P. Peñas-Lledó E. M. (2021). Relationships between CYP1A2, CYP2C9, CYP2C19, CYP2D6 and CYP3A4metabolic phenotypes and genotypes in a Nicaraguan mestizo population. Pharmacogenomics J. 21 (2), 140–151. 10.1038/s41397-020-00190-9 33024249

[B21] de Leon J. (2020). Personalizing dosing of risperidone, paliperidone and clozapine using therapeutic drug monitoring and pharmacogenetics. Neuropharmacology 168, 107656. 10.1016/j.neuropharm.2019.05.033 31150659

[B22] DeLeon A. Patel N. C. Crismon M. L. (2004). Aripiprazole: a comprehensive review of its pharmacology, clinical efficacy, and tolerability. Clin. Ther. 26 (5), 649–666. 10.1016/s0149-2918(04)90066-5 15220010

[B23] Delphin N. Aust C. Griffiths L. Fernandez F. (2024). Epigenetic regulation in schizophrenia: focus on methylation and histone modifications in human studies. Genes 15 (3), 272. 10.3390/genes15030272 38540331 PMC10970389

[B24] Diep U. Chudow M. Sunjic K. M. (2017). Pharmacokinetic changes in liver failure and impact on drug therapy. AACN Adv. Crit. Care 28 (2), 93–101. 10.4037/aacnacc2017948 28592464

[B25] Dong E. Nelson M. Grayson D. R. Costa E. Guidotti A. (2008). Clozapine and sulpiride but not haloperidol or olanzapine activate brain DNA demethylation. Proc. Natl. Acad. Sci. U. S. A. 105 (36), 13614–13619. 10.1073/pnas.0805493105 18757738 PMC2533238

[B26] Dorado P. González I. Naranjo M. E. de Andrés F. Peñas-Lledó E. M. Calzadilla L. R. (2017). Lessons from Cuba for global precision medicine: CYP2D6 genotype is not a robust predictor of CYP2D6 ultrarapid metabolism. Omics 21, 17–26. 10.1089/omi.2016.0166 28271978

[B27] Duan R. Fu Q. Sun Y. Li Q. (2022). Epigenetic clock: a promising biomarker and practical tool in aging. Ageing Res. Rev. 81, 101743. 10.1016/j.arr.2022.101743 36206857

[B28] Fitz-James M. H. Cavalli G. (2022). Molecular mechanisms of transgenerational epigenetic inheritance. Nat. Rev. Genet. 23 (6), 325–341. 10.1038/s41576-021-00438-5 34983971 PMC7619059

[B29] Forster J. Duis J. Butle r M. G. (2021). Pharmacogenetic testing of cytochrome P450 drug metabolizing enzymes in a case series of patients with Prader-Willi syndrome. Genes 12 (2), 152. 10.3390/genes12020152 33498922 PMC7912498

[B30] Guccione E. Schwarz M. Di Tullio F. Mzoughi S. (2021). Cancer synthetic vulnerabilities to protein arginine methyltransferase inhibitors. Curr. Opin. Pharmacol. 59, 33–42. 10.1016/j.coph.2021.04.004 34052526 PMC8759094

[B31] Guidotti A. Auta J. Chen Y. Davis J. M. Dong E. Gavin D. P. (2011). Epigenetic GABAergic targets in schizophrenia and bipolar disorder. Neuropharmacology 60 (7-8), 1007–1016. 10.1016/j.neuropharm.2010.10.021 21074545 PMC4144274

[B32] Habano W. Kawamura K. Iizuka N. Terashima J. Sugai T. Ozawa S. (2015). Analysis of DNA methylation landscape reveals the roles of DNA methylation in the regulation of drug metabolizing enzymes. Clin. Epigenet 7, 105. 10.1186/s13148-015-0136-7 26421064 PMC4587720

[B33] Hakkola J. Hukkanen J. Turpeinen M. Pelkonen O. (2020). Inhibition and induction of CYP enzymes in humans: an update. Arch. Toxicol. 94 (11), 3671–3722. 10.1007/s00204-020-02936-7 33111191 PMC7603454

[B34] Halbert C. H. (2022). Equity in genomic medicine. Annu. Rev. Genomics Hum. Genet. 23, 613–625. 10.1146/annurev-genom-112921-022635 35363547 PMC12520619

[B35] Hasanzad M. Sarhangi N. Naghavi A. Ghavimehr E. Khatami F. Ehsani Chimeh S. (2021). Genomic medicine on the frontier of precision medicine. J. Diabetes Metab. Disord. 21 (1), 853–861. 10.1007/s40200-021-00880-6 35673457 PMC9167337

[B36] He H. Nie Y. L. Li J. F. Meng X. G. Yang W. H. Chen Y. L. (2016). Developmental regulation of CYP3A4 and CYP3A7 in Chinese Han population. Drug Metab. Pharmacokinet. 31 (6), 433–444. 10.1016/j.dmpk.2016.08.008 27727071

[B37] Helsby N. A. Burns K. E. (2012). Molecular mechanisms of genetic variation and transcriptional regulation of CYP2C19. Front. Genet. 3, 206. 10.3389/fgene.2012.00206 23087703 PMC3467616

[B38] Hiemke C. Bergemann N. Clement H. W. Conca A. Deckert J. Domschke K. (2018). Consensus guidelines for therapeutic drug monitoring in neuropsychopharmacology: update 2017. Pharmacopsychiatry 51 (1-02), 9–62. 10.1055/s-0043-116492 28910830

[B39] Imani S. Becatti M. Khan M. A. (2022). Editorial: molecular targeted therapy in oncology: lessons from pharmacogenetics and pharmacoepigenetics. Front. Mol. Biosci. 9, 822188. 10.3389/fmolb.2022.822188 35372509 PMC8964776

[B40] Ingelman-Sundberg M. Sim S. C. (2010). Pharmacogenetic biomarkers as tools for improved drug therapy; emphasis on the cytochrome P450 system. Biochem. Biophys. Res. Commun. 396 (1), 90–94. 10.1016/j.bbrc.2010.02.162 20494117

[B41] Ingelman-Sundberg M. Sim S. C. Gomez A. Rodriguez-Antona C. (2007). Influence of cytochrome P450 polymorphisms on drug therapies: pharmacogenetic, pharmacoepigenetic and clinical aspects. Pharmacol. Ther. 116 (3), 496–526. 10.1016/j.pharmthera.2007.09.004 18001838

[B42] Jin J. Zhong X. B. (2023). Epigenetic mechanisms contribute to intraindividual variations of drug metabolism mediated by cytochrome P450 enzymes. Drug Metab. Dispos. 51 (6), 672–684. 10.1124/dmd.122.001007 36973001 PMC10197210

[B43] Johannessen Landmark C. Johannessen S. I. Patsalos P. N. (2020). Therapeutic drug monitoring of antiepileptic drugs: current status and future prospects. Expert Opin. Drug Metab. Toxicol. 16 (3), 227–238. 10.1080/17425255.2020.1724956 32054370

[B44] Kacevska M. Ivanov M. Wyss A. Kasela S. Milani L. Rane A. (2012). DNA methylation dynamics in the hepatic CYP3A4 gene promoter. Biochimie 94 (11), 2338–2344. 10.1016/j.biochi.2012.07.013 22906825

[B45] Kanu A. A. Johnston M. M. Poweleit E. A. Vaughn S. E. Strawn J. R. Ramsey L. B. (2024). Influence of CYP2D6 metabolizer status on risperidone and paliperidone tolerability in children and adolescents. J. Child. Adolesc. Psychopharmacol. 34 (1), 34–41. 10.1089/cap.2023.0046 38377522

[B46] Kappel D. B. Rees E. Fenner E. King A. Jansen J. Helthuis M. (2024). Rare variants in pharmacogenes influence clozapine metabolism in individuals with schizophrenia. Eur. Neuropsychopharmacol. 80, 47–54. 10.1016/j.euroneuro.2023.12.007 38310750 PMC7619122

[B47] Karnes J. H. Rettie A. E. Somogyi A. A. Huddart R. Fohner A. E. Formea C. M. (2021). Clinical pharmacogenetics implementation Consortium (CPIC) Guideline for CYP2C9 and HLA-B genotypes and phenytoin dosing: 2020 update. Clin. Pharmacol. Ther. 109 (2), 302–309. 10.1002/cpt.2008 32779747 PMC7831382

[B48] Kim D. W. Weon K. Y. Hong E. P. Chung E. K. Lee K. T. (2016). Comparative physicochemical and pharmacokinetic properties of quetiapine and its active metabolite norquetiapine. Chem. Pharm. Bull. 64 (11), 1546–1554. 10.1248/cpb.c16-00223 27803466

[B49] Kolli P. Kelley G. Rosales M. Faden J. Serdenes R. (2023). Olanzapine pharmacokinetics: a clinical review of current insights and remaining questions. Pharmgenomics Pers. Med. 16, 1097–1108. 10.2147/PGPM.S391401 38146514 PMC10749543

[B50] Li Y. (2021). Modern epigenetics methods in biological research. Methods 187, 104–113. 10.1016/j.ymeth.2020.06.022 32645449 PMC7785612

[B51] Li S. Tollefsbol T. O. (2021). DNA methylation methods: global DNA methylation and methylomic analyses. Methods 187, 28–43. 10.1016/j.ymeth.2020.10.002 33039572 PMC7914139

[B52] Li J. Guo Y. Schroeder F. A. Youngs R. M. Schmidt T. W. Ferris C. (2004). Dopamine D2-like antagonists induce chromatin remodeling in striatal neurons through cyclic AMP-Protein kinase A and NMDA receptor signaling. J. Neurochem. 90 (5), 1117–1131. 10.1111/j.1471-4159.2004.02569.x 15312167 PMC4203323

[B53] Lisoway A. J. Chen C. C. Zai C. C. Tiwari A. K. Kennedy J. L. (2021). Toward personalized medicine in schizophrenia: genetics and epigenetics of antipsychotic treatment. Schizophr. Res. 232, 112–124. 10.1016/j.schres.2021.05.010 34049235

[B54] Liu R. Wu J. Guo H. Yao W. Li S. Lu Y. (2023). Post-translational modifications of histones: mechanisms, biological functions, and therapeutic targets. MedComm 4 (3), e292. 10.1002/mco2.292 37220590 PMC10200003

[B55] Maruf A. A. Greenslade A. Arnold P. D. Bousman C. (2019). Antidepressant pharmacogenetics in children and young adults: a systematic review. J. Affect Disord. 254, 98–108. 10.1016/j.jad.2019.05.025 31112844

[B56] Mauri M. C. Paletta S. Di Pace C. Reggiori A. Cirnigliaro G. Valli I. (2018). Clinical pharmacokinetics of atypical antipsychotics: an update. Clin. Pharmacokinet. 57 (12), 1493–1528. 10.1007/s40262-018-0664-3 29915922

[B57] Melka M. G. Laufer B. I. McDonald P. Castellani C. A. Rajakumar N. O'Reilly R. (2014). The effects of olanzapine on genome-wide DNA methylation in the hippocampus and cerebellum. Clin. Epigenetics 6 (1), 1. 10.1186/1868-7083-6-1 24382160 PMC3895844

[B58] Musyoka K. Chan C. W. Gutiérrez Rico E. M. Omondi P. Kijogi C. Okai T. (2024). Genetic variation present in the CYP3A4 gene in Ni-Vanuatu and Kenyan populations in malaria endemicity. Drug Metab. Pharmacokinet. 57, 101029. 10.1016/j.dmpk.2024.101029 39079373

[B59] Narita T. Weinert B. T. Choudhary C. (2019). Functions and mechanisms of non-histone protein acetylation. Nat. Rev. Mol. Cell Biol. 20 (3), 156–174. 10.1038/s41580-018-0081-3 30467427

[B60] Neyshaburinezhad N. Ghasim H. Rouini M. Daali Y. Ardakani Y. H. (2021). Frequency of important CYP450 enzyme gene polymorphisms in the Iranian population in comparison with other major populations: a comprehensive review of the human data. J. Pers. Med. 11 (8), 804. 10.3390/jpm11080804 34442448 PMC8401584

[B61] Ocaña-Paredes B. Rivera-Orellana S. Ramírez-Sánchez D. Montalvo-Guerrero J. Freire M. P. Espinoza-Ferrao S. (2024). The pharmacoepigenetic paradigm in cancer treatment. Front. Pharmacol. 15, 1381168. 10.3389/fphar.2024.1381168 38720770 PMC11076712

[B62] Park H. J. Choi Y. J. Kim J. W. Chun H. S. Im I. Yoon S. (2015). Differences in the epigenetic regulation of cytochrome P450 genes between human embryonic stem cell-derived hepatocytes and primary hepatocytes. PloS one 10 (7), e0132992. 10.1371/journal.pone.0132992 26177506 PMC4503736

[B63] Peñas-Lledó E. M. Llerena A. (2014). CYP2D6 variation, behaviour and psychopathology: implications for pharmacogenomics-guided clinical trials. Br. J. Clin. Pharmacol. 77 (4), 673–683. 10.1111/bcp.12227 24033670 PMC3971983

[B64] Rami F. Z. Nguyen T. B. Oh Y. E. Karamikheirabad M. Le T. H. Chung Y. C. (2022). Risperidone induced DNA methylation changes in dopamine receptor and stathmin genes in mice exposed to social defeat stress. Clin. Psychopharmacol. Neurosci. 20 (2), 373–388. 10.9758/cpn.2022.20.2.373 35466108 PMC9048015

[B65] Recillas-Targa F. (2022). Cancer epigenetics: an overview. Arch. Med. Res. 53 (8), 732–740. 10.1016/j.arcmed.2022.11.003 36411173

[B66] Richa R. Sinha R. P. (2014). Hydroxymethylation of DNA: an epigenetic marker. EXCLI J. 13, 592–610. 26417286 PMC4464262

[B67] Rognoni C. Bertolani A. Jommi C. (2021). Second-generation antipsychotic drugs for patients with schizophrenia: systematic literature review and meta-analysis of metabolic and cardiovascular side effects. Clin. Drug Investig. 41 (4), 303–319. 10.1007/s40261-021-01000-1 33686614 PMC8004512

[B68] Rojas-Macetas A. Medalla-Garro G. Saravia M. Losno R. Valderrama-Wong M. Pariona R. (2023). Potential polymorphic CYP1A2 and CYP2D6-mediated pharmacokinetic interactions between risperidone or olanzapine and selected drugs intended to treat COVID-19. Drug Metab. Bioanal. Lett. 16 (1), 6–13. 10.2174/1872312815666221125112724 36437717

[B69] Saha N. Muntean A. G. (2021). Insight into the multi-faceted role of the SUV family of H3K9 methyltransferases in carcinogenesis and cancer progression. Biochim. Biophys. Acta Rev. Cancer 1875 (1), 188498. 10.1016/j.bbcan.2020.188498 33373647 PMC7856268

[B70] Schoretsanitis G. Spina E. Hiemke C. de Leon J. (2017). A systematic review and combined analysis of therapeutic drug monitoring studies for long-acting risperidone. Expert Rev. Clin. Pharmacol. 10 (9), 965–981. 10.1080/17512433.2017.1345623 28699847

[B71] Shi Y. Li M. Song C. Xu Q. Huo R. Shen L. (2017). Combined study of genetic and epigenetic biomarker risperidone treatment efficacy in Chinese Han schizophrenia patients. Transl. Psychiatry 7 (7), e1170. 10.1038/tp.2017.143 28696411 PMC5538123

[B72] Skadrić I. Stojković O. (2020). Defining screening panel of functional variants of CYP1A1, CYP2C9, CYP2C19, CYP2D6, and CYP3A4 genes in Serbian population. Int. J. Leg. Med. 134 (2), 433–439. 10.1007/s00414-019-02234-7 31858263

[B73] Smigielski L. Jagannath V. Rössler W. Walitza S. Grünblatt E. (2020). Epigenetic mechanisms in schizophrenia and other psychotic disorders: a systematic review of empirical human findings. Mol. Psychiatry 25 (8), 1718–1748. 10.1038/s41380-019-0601-3 31907379

[B74] Smith D. A. Sadler M. C. Altman R. B. (2023). Promises and challenges in pharmacoepigenetics. Camb Prism. Precis. Med. 1, e18. 10.1017/pcm.2023.6 37560024 PMC10406571

[B75] Soria-Chacartegui P. Villapalos-García G. Zubiaur P. Abad-Santos F. Koller D. (2021). Genetic polymorphisms associated with the pharmacokinetics, pharmacodynamics and adverse effects of olanzapine, aripiprazole and risperidone. Front. Pharmacol. 12, 711940. 10.3389/fphar.2021.711940 34335273 PMC8316766

[B76] Spina E. Barbieri M. A. Cicala G. de Leon J. (2020). Clinically relevant interactions between atypical antipsychotics and anti-infective agents. Pharmaceuticals 13 (12), 439. 10.3390/ph13120439 33276675 PMC7761579

[B77] Srivastava A. Dada O. Qian J. Al-Chalabi N. Fatemi A. B. Gerretsen P. (2021). Epigenetics of schizophrenia. Psychiatry Res. 305, 114218. 10.1016/j.psychres.2021.114218 34638051

[B78] Strahl B. D. Allis C. D. (2000). The language of covalent histone modifications. Nature 403 (6765), 41–45. 10.1038/47412 10638745

[B79] Sun X. J. Man N. Tan Y. Nimer S. D. Wang L. (2015). The role of histone acetyltransferases in normal and malignant hematopoiesis. Front. Oncol. 5, 108. 10.3389/fonc.2015.00108 26075180 PMC4443728

[B80] Swathy B. Banerjee M. (2017). Understanding epigenetics of schizophrenia in the backdrop of its antipsychotic drug therapy. Epigenomics 9 (5), 721–736. 10.2217/epi-2016-0106 28470099

[B81] Swathy B. Banerjee M. (2022). Understanding pharmaco-epigenomic response of antipsychotic drugs using genome-wide MicroRNA expression profile in liver cell line. Front. Mol. Neurosci. 15, 786632. 10.3389/fnmol.2022.786632 35392270 PMC8980709

[B82] Thakur A. Parvez M. M. Leeder J. S. Prasad B. (2021). Ontogeny of drug-metabolizing enzymes. Methods Mol. Biol. 2342, 551–593. 10.1007/978-1-0716-1554-6_18 34272706

[B83] Torcal Garcia G. Graf T. (2021). The transcription factor code: a beacon for histone methyltransferase docking. Trends Cell Biol. 31 (10), 792–800. 10.1016/j.tcb.2021.04.001 34016504

[B84] Ur Rasheed M. S. Mishra A. K. Singh M. P. (2017). Cytochrome P450 2D6 and parkinson's disease: polymorphism, metabolic role, risk and protection. Neurochem. Res. 42 (12), 3353–3361. 10.1007/s11064-017-2384-8 28871472

[B85] Vermeir M. Naessens I. Remmerie B. Mannens G. Hendrickx J. Sterkens P. (2008). Absorption, metabolism, and excretion of paliperidone, a new monoaminergic antagonist, in humans. Drug Metab. Dispos. 36 (4), 769–779. 10.1124/dmd.107.018275 18227146

[B86] Vyhlidal C. A. Bi C. Ye S. Q. Leeder J. S. (2016). Dynamics of cytosine methylation in the proximal promoters of CYP3A4 and CYP3A7 in pediatric and prenatal livers. Drug Metab. Dispos. 44 (7), 1020–1026. 10.1124/dmd.115.068726 26772622

[B87] Wang H. Helin K. (2025). Roles of H3K4 methylation in biology and disease. Trends Cell Biol. 35 (2), 115–128. 10.1016/j.tcb.2024.06.001 38909006

[B88] Wang Z. Zang C. Rosenfeld J. A. Schones D. E. Barski A. Cuddapah S. (2008). Combinatorial patterns of histone acetylations and methylations in the human genome. Nat. Genet. 40 (7), 897–903. 10.1038/ng.154 18552846 PMC2769248

[B89] Wang P. Chen S. Wang Y. Wang X. Yan L. Yang K. (2021). The long noncoding RNA hepatocyte nuclear factor 4α antisense RNA 1 negatively regulates cytochrome P450 enzymes in Huh7 cells *via* histone modifications. Drug Metab. Dispos. 49 (5), 361–368. 10.1124/dmd.120.000316 33674270

[B90] Wielandt A. M. Moreno M. Ortiz L. (2022). Uso de la farmacogenética como herramienta de precisión en psiquiatría: hacia una medicina personalizada [Use of pharmacogenetics as a precision tool in psychiatry: towards a personalized medicine]. Rev. Med. Clin. Condes 33 (2), 163–173. 10.1016/j.rmclc.2022.03.007

[B91] Yan L. Wang Y. Liu J. Nie Y. Zhong X. B. Kan Q. (2017). Alterations of histone modifications contribute to pregnane X receptor-mediated induction of CYP3A4 by rifampicin. Mol. Pharmacol. 92 (2), 113–123. 10.1124/mol.117.108225 28546420 PMC5508193

[B92] Yin T. Miyata T. (2011). Pharmacogenomics of clopidogrel: evidence and perspectives. Thromb. Res. 128 (4), 307–316. 10.1016/j.thromres.2011.04.010 21592545

[B93] Yingyuan Lu. Mei Z. Shengju Y. Xiaona D. Zhiyuan Z. Haixu C. (2023). Epigenetic variants of xenobiotic metabolism affect individual differences in antiepileptic drug 3,4-DCPB pharmacokinetic phenotype. J. Chin. Pharm. Sci. 32 (1), 1–16. 10.5246/jcps.2023.01.001

[B94] Zanger U. M. Raimundo S. Eichelbaum M. (2004). Cytochrome P450 2D6: overview and update on pharmacology, genetics, biochemistry. Arch. Pharmacol. 369, 23–37. 10.1007/s00210-003-0832-2 14618296

[B95] Zhang D. Tang Z. Huang H. Zhou G. Cui C. Weng Y. (2019). Metabolic regulation of gene expression by histone lactylation. Nature 574 (7779), 575–580. 10.1038/s41586-019-1678-1 31645732 PMC6818755

[B96] Zhang L. Lu Q. Chang C. (2020). Epigenetics in health and disease. Adv. Exp. Med. Biol. 1253, 3–55. 10.1007/978-981-15-3449-2_1 32445090

[B97] Zhou S. F. Liu J. P. Chowbay B. (2009). Polymorphism of human cytochrome P450 enzymes and its clinical impact. Drug Metab. Rev. 41 (2), 89–295. 10.1080/03602530902843483 19514967

[B98] Zhou X. Y. Hu X. X. Wang C. C. Lu X. R. Chen Z. Liu Q. (2019). Enzymatic activities of CYP3A4 allelic variants on quinine 3-Hydroxylation *in vitro* . Front. Pharmacol. 10, 591. 10.3389/fphar.2019.00591 31214030 PMC6555127

[B99] Zubiaur P. Fernández-Campos P. Navares-Gómez M. Soria-Chacartegui P. Villapalos-García G. Román M. (2021). Variants in COMT, CYP3A5, CYP2B6, and ABCG2 alter quetiapine pharmacokinetics. Pharmaceutics 13 (10), 1573. 10.3390/pharmaceutics13101573 34683865 PMC8540141

